# Dissociating Perceptual Confidence from Discrimination Accuracy Reveals No Influence of Metacognitive Awareness on Working Memory

**DOI:** 10.3389/fpsyg.2016.00851

**Published:** 2016-06-06

**Authors:** Jason Samaha, John J. Barrett, Andrew D. Sheldon, Joshua J. LaRocque, Bradley R. Postle

**Affiliations:** ^1^Department of Psychology, University of Wisconsin–Madison, MadisonWI, USA; ^2^Medical Scientist Training Program and Neuroscience Training Program, University of Wisconsin–Madison, MadisonWI, USA; ^3^Department of Psychiatry, University of Wisconsin–Madison, MadisonWI, USA

**Keywords:** working memory, metacognition, relative blindsight, confidence, perception

## Abstract

Visual awareness is hypothesized to be intimately related to visual working memory (WM), such that information present in WM is thought to have necessarily been represented consciously. Recent work has challenged this longstanding view by demonstrating that visual stimuli rated by observers as unseen can nevertheless be maintained over a delay period. These experiments have been criticized, however, on the basis that subjective awareness ratings may contain response bias (e.g., an observer may report no awareness when in fact they had partial awareness). We mitigated this issue by investigating WM for visual stimuli that were matched for perceptual discrimination capacity (*d*′), yet which varied in subjective confidence ratings (so-called relative blindsight). If the degree of initial subjective awareness of a stimulus facilitates later maintenance of that information, WM performance should improve for stimuli encoded with higher confidence. In contrast, we found that WM performance did not benefit from higher visual discrimination confidence. This relationship was observed regardless of WM load (1 or 3). Insofar as metacognitive ratings (e.g., confidence, visibility) reflect visual awareness, these results challenge a strong relationship between conscious perception and WM using a paradigm that controls for discrimination accuracy and is less subject to response bias (since confidence is manipulated within subjects). Methodologically, we replicate prior efforts to induce relative blindsight using similar stimulus displays, providing a general framework for isolating metacognitive awareness in order to examine the function of consciousness.

## Introduction

When making perceptual decisions, human observers have the capacity to introspect about the quality of their perceptual experience. These metacognitive judgments are often quantified using subjective ratings on confidence or visibility scales (Kanai et al., 2010; Maniscalco and Lau, 2012; Yeung and Summerfield, 2012; Barrett et al., 2013). In most situations, metacognitive awareness is highly coupled with objective decision accuracy such that when accuracy is high perceptual confidence is as well. However, the phenomenon of blindsight, where individuals with damage to primary visual cortex perform above-chance on visual discrimination tasks despite reporting no visual experience, provides compelling evidence that subjective and objective measures of visual awareness can dissociate (Weiskrantz, 1986; Cowey and Stoerig, 1991; Overgaard, 2011; Ko and Lau, 2012; Leopold, 2012). This dissociation has proven useful for studying visual awareness because it may unconfound basic perceptual processes from metacognitive awareness (Rosenthal, 2000; Lau and Rosenthal, 2011).

Attempts to induce blindsight-like phenomena in normal observers have been controversial (Kolb and Braun, 1995; Kunimoto et al., 2001), with initial observations proving difficult to replicate (Morgan et al., 1997; Robichaud and Stelmach, 2003). This difficulty stems, in part, from longstanding complications with subjective reports, going back at least to early criticisms of the introspectionist movement (see Comte as quoted in James, 1890). Namely, it is difficult to prove that observers are *completely* unaware of the stimulus that they are accurately discriminating because their subjective responses may be biased toward underreporting awareness. To mitigate this issue, Lau and Passingham (2006) formulated a paradigm to induce “relative blindsight” in healthy observers. Relative blindsight refers to a comparison between two similar stimulus conditions with comparable objective discrimination accuracy, yet with differing levels of reported awareness. This contrast effectively isolates relative changes in metacognitive awareness, while controlling for task performance, attention, and motivational confounds, which would presumably impact objective accuracy as well (Morales et al., 2015). Furthermore, this approach is less prone to across-subjects response bias in subjective reports because metacognitive awareness is manipulated within each individual (Peters et al., 2016). Although it remains debated whether this approach, which relies on an observer having metacognitive insight (i.e., reportable access to their conscious experience), fully captures all that is present in perceptual experience (Block, 2011; but see Brown, 2014), the subjective feeling of knowing that one has perceived a stimulus often accompanies our visual experience and should be considered an important aspect of visual consciousness.

We sought to use recently developed procedures for generating stimuli that induce relative blindsight in a perceptual two-choice discrimination task, and then use these same stimuli in a working memory (WM) paradigm to test the role of metacognitive awareness in WM maintenance. Two recent reports have demonstrated that one’s perceptual confidence is primarily influenced by the amount of evidence in favor of a stimulus interpretation (e.g., the contrast of an oriented grating; here referred to as “positive evidence”), whereas one’s perceptual capacity (*d*′) relies on the relative amount of positive evidence to evidence against that stimulus interpretation (i.e., the signal-to-noise ratio; Zylberberg et al., 2012; Koizumi et al., 2015). Therefore, manipulating the amount of positive evidence in a stimulus while maintaining the ratio of positive evidence to noise may serve as a robust paradigm for inducing relative blindsight and assessing the relationship between metacognitive awareness and WM.

Theories of consciousness and theories of WM have both posited a close link between these two constructs (reviewed in Soto and Silvanto, 2014). Several WM theorists have focused on whether information *maintained* in WM is conscious or not, and there is agreement that at least attended representations in WM are consciously experienced (Oberauer, 2002; Baddeley, 2003; Cowan, 2011). Empirical and theoretical work on consciousness has focused instead on whether sensory information must be consciously perceived before it can *enter* WM. The strongest version of this claim is expressed in Global Workspace Theory, which posits that neural activation to non-conscious stimuli is relatively short lived and local and may only be maintained over a delay if it becomes consciously available in the global workspace (Dehaene and Naccache, 2001; Baars, 2003; Baars and Franklin, 2003). This relationship has been further supported by the observation that subjective visual awareness and some WM processes may share overlapping neural structures in prefrontal and parietal cortices (Naghavi and Nyberg, 2005; Lau and Passingham, 2006; Cul et al., 2009; Fleming et al., 2010, 2014; Rounis et al., 2010; Persaud et al., 2011; Bor and Seth, 2012; Ester et al., 2015). If a stimulus is perceived with greater metacognitive awareness, it may engage prefrontal and parietal regions to a greater extent, result in sustained neural activity, and may then be made available to WM and other executive functions.

Recent experiments, however, have challenged the claim that metacognitive awareness is necessary for WM (Soto et al., 2011; Bergström and Eriksson, 2014, 2015; Dutta et al., 2014). Across multiple visibility manipulations (backward masking, flash suppression, attentional blink), these authors observed above-chance WM performance on trials for which stimuli were rated as unseen. This line of work has recently been criticized, however, on the basis that subjective reports may be confounded with response bias (Samaha, 2015; Stein et al., 2016). If some subjects are biased toward underreporting awareness of the WM stimulus, then above-chance performance may be due to a degraded, but nevertheless conscious representation of the memoranda. To overcome this limitation, the current study takes a different approach to the question of whether metacognitive awareness is related to WM. Rather than taking the lowest rating on a confidence or visibility scale to truly indicate complete unawareness, we investigate whether relative variation in perceptual confidence predicts variation in subsequent WM performance. We use stimuli that induce relative blindsight (defined from an initial perceptual task) in a WM paradigm to isolate perceptual metacognitive changes while controlling for performance confounds and mitigating across-subject response bias. Our primary interest is in manipulating confidence independently of perceptual discrimination in order to test whether increasing metacognitive awareness, on its own, leads to increased WM performance. Of secondary interest is examining the effect that the perceptual confidence manipulation may have on confidence reports given for WM-based judgments. If the degree of subjective visual awareness determines the extent to which WM representations can be maintained, then WM performance should improve for stimuli encoded with higher perceptual confidence, controlling for perceptual capacity (*d*′). To anticipate, we successfully induced relative blindsight and found that, in contrast to this proposal, higher perceptual confidence had no appreciable effect on WM performance.

## Materials and Methods

### Subjects

Fifteen subjects (9 female, mean age = 20.6 years, *SD* = 1.68) from the University of Wisconsin-Madison community participated in this experiment and received monetary compensation. Sample size was determined based on a power analysis of previously reported effect sizes from three experiments using a nearly identical manipulation (Koizumi et al., 2015). From these three experiments, we averaged the required sample size needed to detect the effect of positive evidence on confidence with 80% power. All subjects provided written consent, reported normal or corrected-to-normal visual acuity and color vision, and were blind to the hypothesis of the experiment. The University of Wisconsin-Madison Institutional Review Board approved the study.

### Stimuli

**FIGURE 1:**
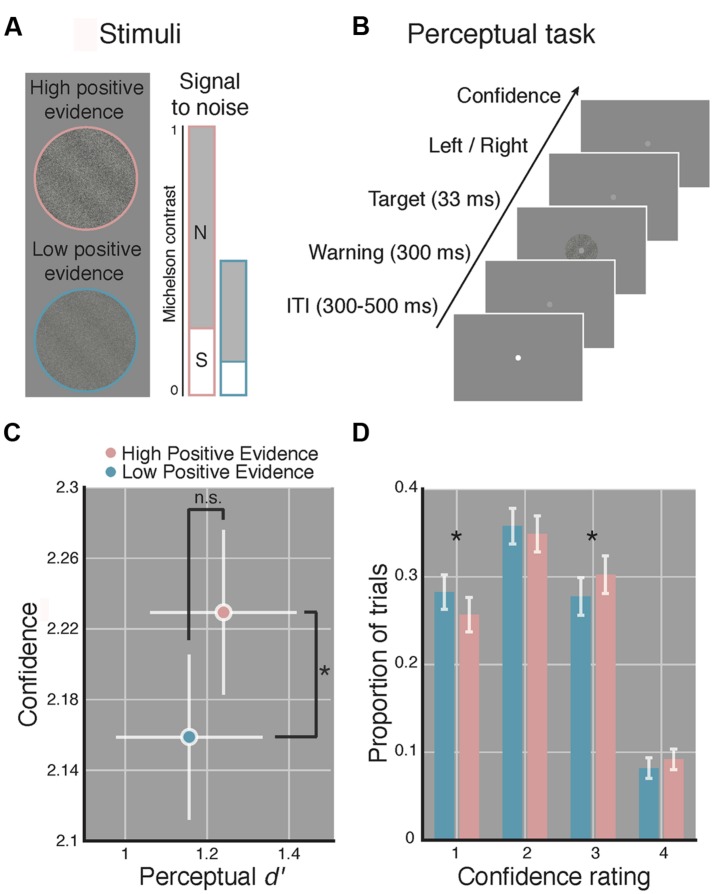
**Manipulating perceptual metacognitive awareness independently of discrimination accuracy.** **(A)** High (pink) and low (blue) positive evidence stimuli were constructed by manipulating the overall contrast of a target grating embedded in noise, while maintaining the same signal-to-noise contrast ratio. Following prior work (Zylberberg et al., 2012; Koizumi et al., 2015), we anticipated the absolute level of positive evidence (target contrast) to influence confidence, yet the ratio of positive evidence to noise to dictate *d*′. Colors not shown on actual displays. **(B)** The perceptual task completed at the beginning and end of the experiment required subjects to make a two-choice discrimination (left or right) based on the tilt of the grating and then rate their confidence on a 1–4 scale (low–high). **(C)** Average *d*′ and confidence across both blocks of the perceptual task. Stimuli with higher positive evidence were reliably judged with higher confidence, despite no significant difference in *d*′ between conditions. **(D)** Effect of positive evidence broken down by the proportion of trials each rating was used. Decreasing positive evidence caused a significant decrease in “3” ratings, and an increase in “1” ratings, suggesting a shift in the distribution of confidence ratings. Asterisk indicates *p* < 0.05, ns denotes non-significant effect. Error bars indicate 95% confidence intervals on the positive evidence effect.

Target stimuli were sinusoidal luminance gratings embedded in random dot noise presented within a circular aperture (see **Figure 1A**). Gratings subtended 2 degrees of visual angle (DVA), had a spatial frequency of 1.5 cycles/DVA, a phase of zero, and were rotated 45 or -45° from vertical. Noise consisted of random black and white pixels. Michelson contrast (Michelson, 1927; defined as the luminance difference between the brightest and dimmest pixel of the stimulus, divided by their sum) was computed for both signal (the grating) and the noise. We use the term positive evidence to refer to the contrast of the grating signal and the term noise to refer to the contrast level of the noise. Following Zylberberg et al. (2012) and Koizumi et al. (2015), who found that, across multiple types of stimuli, perceptual confidence was driven by the absolute value of positive evidence in favor of a perceptual decision, whereas *d*′ was driven by the ratio of positive to negative evidence/noise, we created two sets of grating stimuli with differing levels of positive evidence (high or low) but with equal ratios of positive evidence (signal) to noise (see **Figure 1A**). The ratio of positive evidence to noise used for the high positive evidence condition was first determined for each subject by an adaptive staircase procedure (see Procedure). We then halved the contrast of both positive evidence and noise to create the low positive evidence condition while maintaining the same ratio of positive evidence to noise.

**FIGURE 2:**
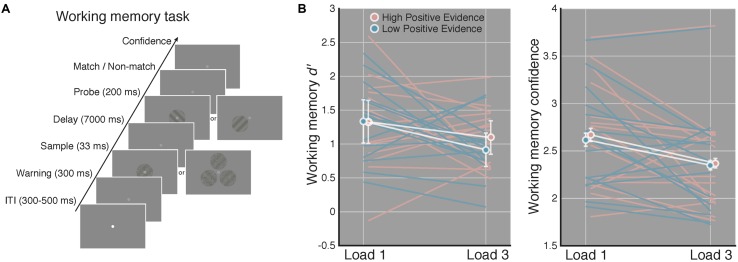
**Increased perceptual confidence does not translate to improved WM performance.** **(A)** After completing the perceptual task in **Figure 1**, subjects completed a WM task using the same high and low positive evidence stimuli from the perceptual task. Load (1 or 3) was manipulated across blocks and subjects were asked to indicate whether a highly visible probe orientation matched or did not match the orientation of the sample grating that previously occupied the same screen location 7 s prior. They then rated their confidence from 1 to 4 (low–high). This example is of a non-match trial. **(B)** Match/non-match *d*′ and average confidence ratings as a function of load and positive evidence conditions. We observed significantly lower *d*′ and confidence on load 3 as compared to load 1 trials, but no effect of positive evidence on *d*′ or confidence, and no interaction with load. This suggests that greater perceptual confidence (**Figure 1**) did not lead to better WM performance. White lines denote condition means and blue and pink lines represent individual subjects, error bars indicate 95% confidence intervals on the positive evidence difference.

Stimuli were presented on an iMac computer screen (52 cm wide × 32.5 cm tall; 1920 × 1200 resolution; 60 Hz refresh rate). Subjects viewed the screen from a chin rest at a distance of 62 cm. Stimuli were generated and presented using the MGL toolbox^1^ running in MATLAB 2015b (MathWorks, Natick, MA, USA). Fixation (a light gray point, 0.08 DVA) was centered on the screen and stimuli were presented at fixation against a gray screen background. When three gratings were presented on load 3 WM trials these appeared evenly spaced along a concentric path around fixation, with each grating centered 1.25 DVA away from fixation (see **Figure 2A**).

### Procedure

Over the course of a single 1.5-h testing session, subjects performed a staircase task, then a perceptual task, then a WM task, and then more of the perceptual task (total of 670 trials). For the perceptual task, subjects were instructed to report the orientation (left or right) of a target grating and then rate their confidence in their decision on a 1–4 scale (low–high). For the WM task, subjects indicated whether a highly visible probe grating matched or did not match the orientation of the target grating that previously occupied the same screen location (see **Figure 2A**), followed by a confidence report. Task order was as follows: first, each subject completed 110 trials of a 1-up, 3-down staircase procedure, controlled with the PEST algorithm (Taylor and Creelman, 1967), intended to produce ∼ 79% discrimination accuracy. During the staircase, the Michelson contrast of the target grating was manipulated relative to noise contrast, which was was set to 1 and did not change. The first 10 trials were considered practice and were non-adaptive with highly visible gratings. Subjects rated confidence after each trial during the staircase to become familiar with the scale but we did not analyze these data. The contrast defined by averaging the last 10 reversals of the staircase was used as the high positive evidence condition and half this contrast value (and half the noise contrast) defined the low positive evidence condition (see Stimuli). Next, subjects completed 160 trials of a perceptual task (shown in **Figure 1B**) wherein condition (low or high positive evidence) and grating orientation (left or right) were randomly determined (with replacement) on each trial. Following an inter-trial interval of random duration between 300 and 500 ms, the fixation point dimmed to warn subjects of the start of the trial and 300 ms later the target grating appeared (for a duration of 33 ms). This tasks was used to test whether we could dissociate perceptual confidence from discrimination accuracy (*d*′) and thus replicate Koizumi et al. (2015) with slightly different stimuli.

After the perceptual task, subjects completed four blocks of a match/non-match WM task using the same low and high positive evidence stimuli. Each block was 60 trials long and subjects either had to remember 1 (“load 1”) or 3 (“load 3”) orientations, depending on the block. Because previous work has examined the relationship between awareness and WM using only one or two stimuli (e.g., Soto et al., 2011), we included load 3 trials to assess whether subjective awareness may improve WM when a greater number of items are to be maintained. Block order was interleaved and counterbalanced across subjects. Within a block, positive evidence condition and grating orientation were randomly determined on each trial. For any given load 3 trials, each of the 3 stimuli contained the same low or high positive evidence, but their orientations were chosen randomly. On 50% of trials, the probe grating matched the target grating; on the remaining 50% of trials the probe grating was opposite the orientation of the target (e.g., 45 or -45°). Stimulus timing was the same as in the perceptual task except that a 7-s delay period elapsed prior to the onset of the probe grating, which was displayed for 200 ms.

Following the four WM blocks, subjects completed another 160 trials of the perceptual task in order to better estimate perceptual *d*′ and confidence across the entire experiment. Across all tasks subjects were given unlimited time to respond and were instructed to emphasize accuracy over speed of their responses. Responses were made on a computer keyboard using the left and right arrow keys to indicate grating orientation or match/non-match decision (with the subject’s right hand), and using numerical keys 1–4 to indicate confidence (with the subject’s left hand).

### Data Analysis

*d*′ was calculated separately for each positive evidence condition (low and high) and for each task and WM load using the standard signal detection theory (Stanislaw and Todorov, 1999): *d*′ was computed as *z(hit rate)–z(false alarm rate)*, and criterion (bias) was defined as -*0.5 × (z(hit rate) + z(false alarm rate))*, where *z* denotes the inverse of the normal cumulative distribution function. Confidence ratings were averaged across each condition. Confidence and *d*′ were computed separately for the first and second blocks of the perceptual task and were averaged to provide an estimate most reflective of perception across the entire experiment. Paired-sample *t*-tests were used to assess the effect of positive evidence on *d*′ and confidence from the perceptual task. Confidence and *d*′ from the WM task were independently analyzed with a 2 (positive evidence: low or high) by 2 (load: 1 or 3) repeated-measure ANOVA.

The manipulation of the amount of positive evidence was intended to affect confidence while leaving discrimination accuracy unaffected. However, establishing a null effect through traditional statistical hypothesis testing is problematic because a non-significant statistical test could indicate either no effect or a lack of statistical power. Therefore, we planned to calculate Bayes factors to evaluate the relationship between positive evidence level and perceptual and WM *d*′, using a calculator freely available online^2^. A Bayes factor is a ratio of the evidences supporting two different hypotheses, both of which must be defined. The first hypothesis to be compared is the null hypothesis, that the effect of positive evidence on discrimination accuracy is precisely zero. To define the alternative hypothesis, we looked to Koizumi et al. (2015), who used very similar stimuli and analyses to the present study. In their Experiment 2a, there was a significant interaction between positive evidence and task difficulty, driven by the effect of positive evidence on *d*′ in the “difficult” condition. We reasoned that if we were to see any non-zero effect of positive evidence on perceptual *d*′ or WM *d*′ in our study, it might be of similar magnitude to that seen in Koizumi et al. (2015). Therefore, we defined the alternative hypothesis with a normal distribution centered at the value from Koizumi et al. (2015), with a standard deviation set to one half the mean so that the distribution would not substantially include values below zero (which would be in the opposite direction of the predicted effect, per Dienes, 2014). We calculated the Bayes factor as the ratio of evidence for the alternative hypothesis to the evidence supporting the null hypothesis, so that values less than one favor the null hypothesis and values greater than one favor the alternative hypothesis.

Although subjects were instructed to prioritize accuracy over speed of their responses, we also analyzed response times (RTs) to assess whether manipulating positive evidence impacted a metric of performance that may be sensitive to different aspects of processing than *d*′. Trials with RTs exceeding ± 3 *SD* of the mean of each condition were excluded and RTs were log-transformed, prior to averaging across trials, to adjust for the strong positive skew of the data. (Note that we report mean RTs prior to log-transformation for ease of interpretation.) We additionally analyzed median RTs. Paired-sample *t*-tests were used to compare the effects of evidence on RTs during the perceptual task and a 2 (positive evidence: low or high) by 2 (load: 1 or 3) repeated-measure ANOVA was used on RTs from the WM task.

## Results

### Perceptual Task

In line with prior findings (Zylberberg et al., 2012; Koizumi et al., 2015), we observed significantly higher confidence ratings for stimuli with high, as compared to low positive evidence [*t*(1,14) = 3.07, *p* = 0.008], yet we detect no reliable difference in *d*′ [*t*(1,14) = 0.95, *p* = 0.36; see **Figure 1C**]. The Bayes factor for the effect of positive evidence on *d*′ was 0.11, favoring the interpretation that the non-significant *p*-value is truly due to a null effect (specifically, this indicates that the null is 9 times more likely than the alternative). Analysis of RTs revealed a reliable effect of positive evidence [*t*(1,14) = 7.24, *p* < 0.001; *M*_low_ = 908 ± 65 ms, *M*_high_ = 877 ± 64 ms], indicating faster responses for stimuli with high positive evidence, mirroring with the effects of positive evidence on confidence. This relationship was observed when using median RTs as well [*t*(1,14) = 5.04, *p* < 0.001]. Analysis of criteria showed no differences between high and low positive evidence [*t*(1,14) = 0.57, *p* = 0.572], suggesting that subjects were not reliably more biased toward responding with a particular orientation as a function of positive evidence.

To better understand how the average difference in perceptual confidence that we observed translates to subjects’ use of the confidence scale, we analyzed the proportion of trials on which each confidence rating was used as a function of the level of positive evidence (see **Figure 1D**). Paired-sample *t-*tests at each confidence level indicate that reducing positive evidence led to a reduced frequency of “3” responses and an increased frequency of “1” responses (*p-*value per confidence rating: *p*_conf1_ = 0.024, *p*_conf2_ = 0.41, *p*_conf3_ = 0.044, *p*_conf4_ = 0.11). This suggests that low positive evidence was associated with a leftward shift of the distribution of confidence scores, resulting in reliable pairwise changes at levels 3 and 1. Together, these results indicate that our stimuli reliably induced relative blindsight. On the basis of these findings, we use these stimuli to make inferences about the role of metacognitive awareness in WM, assuming comparable perceptual encoding of the same stimuli during WM.

### Working Memory Task

The ANOVA on WM *d*′ revealed a significant main effect of load [*F*(1,14) = 6.04, *p* = 0.027], indicating worse performance on load 3 compared to load 1 trials. Of primary relevance to our study, we did not observed a significant main effect of positive evidence on WM *d*′ [*F*(1,14) = 1.78, *p* = 0.203] or an interaction of positive evidence with load [*F*(1,14) = 0.54, *p* = 0.472]. Further, for load 1 WM trials, which are most analogous to the perceptual task in that only a single stimulus was displayed, mean *d*′ was virtually identical on high (*M* = 1.32) and low (*M* = 1.33) positive evidence trials [*t*(1,14) = 0.04, *p* = 0.97]. The paired contrast at load 3 trials was also non-significant [*t*(1,14) = 1.45, *p* = 0.167]. These non-significant results were supported to different degrees by the corresponding Bayes factors. At load 1, the Bayes factor was 0.10, indicating strong evidence for the null hypothesis (specifically, that the null is 10 times more likely than the alternative). At load 3, the Bayes factor was 0.48, indicating a more marginal level of evidence that nonetheless still favors the null hypothesis, by a factor of 2, over the alternative. Together, these results suggest that higher positive evidence, which gave rise to more confident perceptual decisions, did not lead to improved WM performance on either load 1 or load 3 trials (see **Figure 2A**).

The analysis of WM confidence showed a significant main effect of load [*F*(1,14) = 5.08, *p* = 0.040], indicating that subjects reported lower confidence on load 3 trials, mirroring the effect of load on *d*′. In contrast to the effect of positive evidence on confidence during the perceptual task, however, we did not observe a significant main effect of positive evidence on WM confidence [*F*(1,14) = 2.35, *p* = 0.14], although confidence tended to be higher for high positive evidence (*M*_load1_ = 2.67, *M*_load3_ = 2.37), than for low positive evidence (*M*_load1_ = 2.62, *M*_load3_ = 2.35; see **Figure 2B**). RTs followed this same pattern, showing a main effect of load [*F*(1,14) = 26.42, *p* < 0.001; *M*_lowLoad1_ = 1628 ± 104 ms; *M*_highLoad1_ = 1589 ± 82 ms, *M*_lowLoad3_ = 2094 ± 99 ms, *M*_highLoad3_ = 1987 ± 91 ms], but no main effect of evidence (*p* = 0.17) and no interaction (*p* = 0.31). Using median RTs revealed the same pattern, with a significant effect of load [*F*(1,14) = 31.35, *p* < 0.001], but a non-significant effect of evidence (*p* = 0.53) and no interaction (*p* = 0.63). The analysis of criteria revealed no main effects or interactions (all *p*-values > 0.28), indicating that neither load nor amount of positive evidence reliably biased subjects toward a certain response (i.e., match or non-match). Removing a single subject who performed at chance on load 1 WM high positive evidence trials did not change the significance of any of the above-mentioned results. Together, these results suggest that perceptual confidence had no influence on either WM accuracy or reaction time.

## Discussion

The aim of the present study was to test whether manipulating subjective confidence ratings independently of perceptual discrimination capacity leads to comparable changes in how well information is maintained in WM. In line with previous work (Zylberberg et al., 2012; Koizumi et al., 2015), we found that perceptual confidence depended on the amount of evidence in favor of a perceptual decision, yet discrimination accuracy was driven by the ratio of positive evidence to noise (see **Figure 1**). This result confirmed that our stimuli produced different levels of perceptual confidence, but comparable *d*′. We then used the same stimuli as the memoranda in a WM task. Surprisingly, stimuli that were perceptually encoded with greater subjective confidence were neither better maintained over a 7-s delay period, nor was RT speeded, irrespective of WM load. These results challenge the claim that the degree of conscious perception influences the extent to which information gains access to WM (Dehaene and Naccache, 2001; Baars, 2003; Baars and Franklin, 2003). More generally, this finding is in line with recent work suggesting that many high-level cognitive control functions, such as task preparation and response inhibition may be independent of metacognitive awareness (Lau and Passingham, 2007; van Gaal et al., 2008, 2010; Koizumi et al., 2015). This is perhaps surprising given that similar frontal structures are associated with cognitive control, WM, and metacognitive awareness (Naghavi and Nyberg, 2005; Cul et al., 2009; Persaud et al., 2011; Bor and Seth, 2012; Fleming and Dolan, 2012; Miller and Buschman, 2013; Ester et al., 2015).

A possible explanation for our finding that perceptual confidence did not improve WM maintenance is that, whereas metacognitive awareness may depend on frontal cortex (Cul et al., 2009; Rounis et al., 2010), visual WM maintenance may be subserved principally by sensory regions (Serences et al., 2009; Riggall and Postle, 2012; LaRocque et al., 2014; D’Esposito and Postle, 2015). Thus, WM may only benefit from metacognitive awareness when operations involving frontal cortex are required. This is in line with a recent experiment (Maniscalco and Lau, 2015) that showed that WM for letter strings interfered with metacognitive sensitivity during a concurrent perceptual task only when demanding mental manipulation of the letters was required (when subjects had to alphabetize them). Because manipulation of information in WM may depend on frontal activity (Petrides and Milner, 1982; Petrides, 1995; D’Esposito et al., 1999; Postle, 2005), it is plausible that WM would be improved by initial perceptual confidence when a task requires WM manipulation. This remains to be tested. Simple maintenance, however, as required by our task, does not seem to benefit from perceptual metacognitive awareness.

As the logic of our design rests on the assumption that the perceptual experience underlying the confidence judgments in the perceptual task is comparable to the perceptual experience of the stimulus during the WM task, it is interesting that confidence ratings made during the WM task did not reliably change as a function of positive evidence. This suggests that, perhaps for our stimuli, the positive evidence manipulation does not effectively dissociate WM confidence from WM *d*′, as it does perceptual confidence from perceptual *d*′. Interestingly, this pattern of confidence across the two tasks was mirrored in RTs. Subjects were faster to respond to stimuli with high positive evidence in the perceptual task, but not in the WM task. This is consistent with longstanding proposals that confidence judgments are an inverse linear function of RTs (Volkmann, 1934), and further supports a distinction between perceptual-based and WM-based confidence reports. Several recent experiments have proposed that distinct metacognitive systems and behavior may exist for perceptual as compared to memory-based judgments, with memory-based metacognition sometimes being more accurate (Fleming et al., 2014) and relating more to parietal structures, as compared to perceptual metacognition, which may be more strongly associated with anterior frontal structures (Baird et al., 2013; McCurdy et al., 2013; Fleming et al., 2014). This may also relate to the observation that, in the present data, confidence ratings tended to be higher overall during the WM task. This is possibly due to having additional deliberation time (i.e., the delay period) during which to form a confidence judgment. Although we cannot be absolutely certain that subjects’ perceptual experience of the same stimulus was equivalent across our two tasks, we believe that differences between tasks, which could entail different mechanisms of making a metacognitive judgment, is the most likely reason why our manipulation of positive evidence affected perceptual confidence, but not WM-based confidence.

Finally, as our task was designed to assess the impact of perceptual confidence on the passage of information into WM, it remains unclear how consciousness is involved when stimuli are already in WM. Many theories propose that information in WM is consciously represented so long as it is in the focus of attention (Oberauer, 2002; Baddeley, 2003; Cowan, 2011), while others propose that attentional state changes alone cannot account for certain differences between WM information content and the way that WM content is experienced or introspected upon (Jacob et al., 2015; Jacobs and Silvanto, 2015). In this respect, maintenance of the stimuli in our task may transpire through a conscious representation of an initial perceptual decision or guess. Future work will need to probe metacognitive awareness during maintenance to understand how WM content is experienced. Nevertheless, insofar as perceptual metacognition is reflective of conscious experience, our results challenge the notion that visual awareness influences subsequent WM performance. We do so using a paradigm that is less influenced by response bias, and controls for perceptual capacity confounds. Furthermore, we validate the relative blindsight paradigm introduced by Koizumi et al. (2015) as a robust paradigm for selectively manipulating perceptual metacognition in order to provide insight into the behavioral consequences of visual awareness.

## Author Contributions

JS, JL, and BRP conceived the study; JS and AS designed the task; JB and AS collected the data; JS and JL analyzed the data; JS drafted the article with assistance from JL; JS, JB, AS, JL, and BP revised and approved the manuscript.

## Conflict of Interest Statement

The authors declare that the research was conducted in the absence of any commercial or financial relationships that could be construed as a potential conflict of interest.

## Footnotes

1 http://gru.stanford.edu

2 http://www.lifesci.sussex.ac.uk/home/Zoltan_Dienes/inference/Bayes.htm
